# Bortezomib Does Not Reduce Muscular Dystrophy in the *dy^2J^/dy^2J^* Mouse Model of Laminin α2 Chain-Deficient Muscular Dystrophy

**DOI:** 10.1371/journal.pone.0146471

**Published:** 2016-01-05

**Authors:** Zandra Körner, Madeleine Durbeej

**Affiliations:** Muscle Biology Unit, Department of Experimental Medical Science, Lund University, Lund, Sweden; Rutgers University -New Jersey Medical School, UNITED STATES

## Abstract

Congenital muscular dystrophy with laminin α2 chain-deficiency, also known as MDC1A, is a severe neuromuscular disorder for which there is no cure. Patients with complete laminin α2 chain-deficiency typically have an early onset disease with a more severe muscle phenotype while patients with residual laminin α2 chain expression usually have a milder disease course. Similar genotype-phenotype correlations can be seen in the *dy*^*3K*^*/dy*^*3K*^ and *dy*^*2J*^*/dy*^*2J*^ mouse models of MDC1A, respectively, with *dy*^*3K*^*/dy*^*3K*^ mice presenting the more severe phenotype. Recently, we demonstrated that the proteasome inhibitor bortezomib partially improves muscle morphology and increases lifespan in *dy*^*3K*^*/dy*^*3K*^ mice. Here, we explore the use of bortezomib in *dy*^*2J*^*/dy*^*2J*^ animals. However, bortezomib neither improved histological hallmarks of disease nor increased muscle strength and locomotive activity in *dy*^*2J*^*/dy*^*2J*^ mice. Altogether our data suggest that proteasome inhibition does not mitigate muscle dysfunction caused by partial laminin α2 chain-deficiency. Still, it is possible that proteasome inhibition could be useful as a supportive therapy in patients with complete absence of laminin α2 chain.

## Introduction

Mutations in the *LAMA2* gene, encoding the laminin α2 chain of the extracellular matrix protein laminin-211, leads to congenital muscular dystrophy type 1A (MDC1A), which is a life threatening disease. Genotype-phenotype analyses have demonstrated that complete deficiency of laminin α2 chain causes a more severe phenotype whereas partial absence leads to a milder disease course. The clinical manifestations of complete laminin α2 chain-deficiency include profound hypotonia at birth, widespread muscle weakness, proximal joint contractures, scoliosis, elevated serum creatine kinase levels and delayed motor milestones. Patients may achieve unsupported sitting but very few children acquire independent ambulation. Individuals with partial laminin α2 chain deficiency often have later onset of proximal muscle weakness and delayed motor milestones but achieve independent ambulation. Histologically, MDC1A skeletal muscle displays typical dystrophic characteristics with degenerating/regenerating and atrophic fibers, early inflammation and extensive connective tissue infiltration [1–4].

There are several mouse models for laminin α2 chain-deficiency that represent the clinical heterogeneity of MDC1A and phenocopy the skeletal muscle changes. The *dy*^*3K*^*/dy*^*3K*^ mouse completely lacks laminin α2 chain and displays a very severe muscular dystrophy and peripheral neuropathy with a median survival of 22 to 23 days. The *dy*^*2J*^*/dy*^*2J*^ mouse model, on the other hand, has slightly reduced expression of a truncated laminin α2 chain that lacks the N-terminal domain involved in laminin polymerization. Consequently, *dy*^*2J*^*/dy*^*2J*^ mice show a relatively mild muscular dystrophy (but with a severe peripheral neuropathy) and live more than six months [5–8].

Importantly, analyses of *dy*^*3K*^*/dy*^*3K*^ and *dy*^*2J*^*/dy*^*2J*^ mice and other MDC1A mouse models have identified several disease driving mechanisms for MDC1A. For example, we have previously shown that there is increased proteasome activity in human MDC1A myoblasts and myotubes. We have also demonstrated significantly enhanced expression of proteasome-related genes and proteins both in d*y*^*3K*^*/dy*^*3K*^ as well as in *dy*^*2J*^*/dy*^*2J*^ muscle, although the increase was more profound in d*y*^*3K*^*/dy*^*3K*^ compared to *dy*^*2J*^*/dy*^*2J*^ mice [9, 10]. Moreover, administration of the proteasome inhibitors MG-132 and bortezomib, respectively, partially improved muscular dystrophy in d*y*^*3K*^*/dy*^*3K*^ mice. Bortezomib improved histological hallmarks of disease, enhanced body weight, locomotion and survival and partially normalized miRNA expression and reduced the proteasome activity in human MDC1A myoblasts and myotubes [9, 10]. In order to evaluate if bortezomib also has beneficial effects in the mouse model of partial laminin α2 chain-deficiency we herein explored the use of bortezomib in *dy*^*2J*^*/dy*^*2J*^ mice. Quite unexpectedly, we found that bortezomib did not ameliorate any of the muscular dystrophy features in the *dy*^*2J*^*/dy*^*2J*^ mouse model.

## Materials and Methods

### Transgenic Animals

Heterozygous *dy*^*2J*^*/dy*^*2J*^ mice (B6.WK-Lama2dy-2J/J) were purchased from the Jackson Laboratory (Bar Harbor, ME) and bred and maintained in the animal facilities of the Biomedical Center at Lund University according to institutional animal care guidelines. Permission was given by the Malmö/Lund (Sweden) ethical committee for animal research (ethical permit numbers M152-14 and M180-14).

### Bortezomib Treatment

Bortezomib was purchased from LC Laboratories (Woburn, MA). A stock solution was stored in -80°C (dissolved in dimethylsulfoxide) and further diluted in sterile sodium chloride before administration. Mice were either injected twice with approximately 0.4 mg/kg i.v. at 2.5 weeks of age and 0.3 mg/kg i.v. at 3.5 weeks of age, or injected altogether six times with approximately 0.4 mg/kg i.v. at 2.5 weeks of age; 0.3 mg/kg i.v. at 3.5 weeks of age; 0.2 mg/kg i.v. at 4.5 weeks of age; 0.2 mg/kg s.c. at 5.5 and 6.5 weeks of age and finally 0.16 mg/kg s.c. at 7.5 weeks of age. Mice were subsequently analyzed at 5.5 and 8.5 weeks of age, respectively. Quadriceps and triceps muscles were processed for immunofluorescence, morphometric analysis and hydroxyproline assay. Plasma was also collected for analysis of circulating miRNAs and creatine kinase activity.

### Histology and Immunofluorescence

Quadriceps and triceps muscle from *dy*^*2J*^*/dy*^*2J*^, bortezomib-treated *dy*^*2J*^*/dy*^*2J*^, wild-type (WT) and bortezomib-treated WT mice were dissected after euthanasia and frozen in optimal cutting temperature compound (Tissue-Tek OCT; Sakura Finetek, Torrance, CA) in liquid nitrogen. Transverse cryosections of 7 μm were stained with hematoxylin and eosin (H&E), Masson’s trichrome (using an HT15 commercial kit; Sigma-Aldrich, St. Louis, MO) or biotinylated wheat germ agglutinin (WGA), which was detected with fluorescein avidin D (Vector Laboratories, Burlingame, CA). Sections were also processed for immunofluorescence analyses according to standard procedures with rat monoclonal anti-tenascin-C (MTn15) [11] and rat monoclonal anti-CD11b (M1/70, BD Pharmingen, San Diego, California). Anti-tenascin-C, anti-CD11b and WGA stained sections were analyzed and images captured with a Zeiss Axioplan fluorescence microscope (Carl Zeiss Microscopy, Jena, Germany) using an ORCA 1394 ER digital camera (Hamamatsu Photonics, Hamamatsu City, Japan) and Openlab software version 3 (Improvision, Coventry, UK).

### Morphometric Analysis

Quantifications were performed on cross sections of entire quadriceps and triceps muscle. H&E and Masson’s trichrome stained sections were scanned using an Aperio ScanScope CS2 scanner with ScanScope console version 8.2.0.1263 (Aperio, Vista, CA). For quantification of tenascin-C and CD11b labeling, we used multiple Tiff-format images at x 10 magnification covering the whole muscle. The area within muscle corresponding to Masson’s trichrome-positive area and to tenascin-C and CD11b labeling was quantified relative to the entire area of the quadriceps and triceps cross section. Images were converted to 8-bit-mode images and the measurements were set to a threshold that was manually adjusted for every individual image (the total muscle area versus stained area, measured in square pixels). The images were analyzed using ImageJ software version 1.43u (NIH, Bethesda, MD). Central nucleation was also quantified using ImageJ. The fiber area of biotinylated WGA stained muscle fibers was measured and quantified using Adobe Photoshop CS5 extended version (Adobe Systems, San Jose, CA).

### Hydroxyproline Assay

OCT blocks with quadriceps and triceps muscle were thawed and washed in PBS. The muscles were weighed and incubated overnight in 200 μl concentrated HCl (12 M) at 95°C. Twenty five μl of hydrolyzate was neutralized with 25 μl NaOH (6 M) and incubated with 450 μl chloramine-T reagent (0.056 M) at room temperature for 25 min. A volume of 500 μl freshly prepared Ehrlich’s reagent [1 M 4-(dimethylamino)benzaldehyde] was added to each sample and incubated at 65°C for 1 h. After cooling on ice, 100 μl in duplicates was transferred to a 96-well plate and absorbance was read at 560 nm. Standards from 4-hydroxyproline at concentrations (μg/ml; 0, 0.05, 0.1, 0.2, 0.4, 0.6, 0.8 and 1.0) were treated the same way as the samples. Absorbance (A_560_) of standards was plotted against amount of hydroxyproline (μg) and a linear regression was performed to determine slope and intercept. All absorbance values were subtracted with blank (0μg/ml hydroxyproline). Content of hydroxyproline in samples was calculated by equation:

$$\text{x}\;(\mu \text{g})\;=\;\left({\text{A}}_{560}-{\text{Y}}_{\text{axisintercept}}\right)/\text{slope}$$

Collagen conversion factor = 13.5 [12, 13]. Values are presented as relative amount of collagen.

### Exploratory Locomotion Test

Exploratory locomotion was evaluated in an open-field test. In each experiment, a mouse was placed into a new cage and allowed to explore the cage for 5 minutes. The time that the mouse spent moving around was measured, as well as number of stand-ups (on hindlimbs).

### Grip Strength

Forelimb grip strength was measured on a grip-strength meter (Columbus Instruments, Columbus, OH) as previously described [14]. In short, the mouse was held by the base of the tail and allowed to grasp the flat wire mesh of the pull bar with its forepaws. When the mouse got a good grip it was slowly pulled away by its tail until it released the pull bar. Each mouse was allowed to pull the pull bar five times. The two lowest values were rejected and the mean of the three remaining values was counted. Animals were not subjected to any training prior to the experiment.

### RNA Isolation from Plasma, cDNA Synthesis and qPCR for miRNA Detection

As previously described [12], blood was collected from heart puncture and transferred to anticoagulant tubes (EDTA), which were centrifuged at 1100 x g for 10 min in 4°C. Total RNA from blood plasma was extracted using the manufacturer’s (miRNeasy Serum/Plasma kit; Qiagen, Valencia, CA) instructions. Briefly, the samples were thawed on ice and then centrifuged at 3000 x g for 5 min in 4°C. Fifty μl plasma was transferred to a new microcentrifuge tube containing 190 μl of QIAzol mixture containing 0.8 μg/μl MS2 bacteriophage RNA (Roche Applied Science, Penzberg, Germany) and incubated for 5 min. Fifty μl of chloroform was added to each tube and incubated for 2 min followed by centrifugation at 12000 x g for 15 min in 4°C. The supernatant was transferred to a new microcentrifuge tube and 435 μl ethanol was added to each sample. The sample was transferred to a spin column, then a rinse step was performed with 1 x 700 μl RWT buffer, 1 x 500 μl RPE buffer and 1 x 500 μl 80% ethanol. Total RNA was eluted by adding 14 μl RNase-free water to the membrane followed by centrifugation at 12000 g for 1 min. The RNA was stored at -80°C. Two μl of eluted blood plasma RNA was reversed transcribe in a 15 μl reaction using the TaqMan MicroRNA Reverse Transcription Kit (Applied Biosystems, Waltham, MA). Two μl of cDNA was assayed in 20 μl PCR reaction according to the protocol for the TaqMan Fast Advanced Master Mix. The amplification was performed in 96-well plates in a LightCycler 480 qPCR system (Roche Diagnostics, Basel, Switzerland). The determination of C_T_ (by the second-derivative method) was done using the manufacturer’s LightCycler software. MiRNA levels were calculated relative to miR-122. Primers/Probes for miR-1, miR-133a and miR-122 were designed by Applied Biosystems (assay ID 002246, 002222 respectively 002245).

### Creatine kinase assay

Blood was collected from heart puncture and transferred to anti-coagulant tubes (EDTA) and centrifuged at 1100 x *g* for 10 min at 4°C. Plasma was analyzed at Clinical Chemistry Laboratory at Skåne University Hospital. The CK_P_S Cobas method was used to quantify enzyme activity.

### Statistical Analysis

Data were analyzed using the Kruskal-Wallis test with a Dunn’s multiple comparison test to determine differences between groups followed Mann-Whitney U-test to determine the differences between two respective groups. Statistical significance was accepted for *P* < 0.05.

## Results and Discussion

### Two bortezomib injections do not reduce muscular dystrophy in *dy*^*2J*^*/dy*^*2J*^ mice

In our previously published study, we demonstrated that *dy*^*3K*^*/dy*^*3K*^ mice injected twice with bortezomib had higher body weight, were more active in an exploratory locomotion test, survived longer and displayed improved muscle morphology compared to non-treated *dy*^*3K*^*/dy*^*3K*^ mice [10]. In order to analyze whether bortezomib has beneficial effects in *dy*^*2J*^*/dy*^*2J*^ mice as well, we injected *dy*^*2J*^*/dy*^*2J*^ mice with bortezomib using a similar administration schedule. Thus, wild-type and *dy*^*2J*^*/dy*^*2J*^ mice were i.v. injected (0.3–0.4 mg/kg) at 2.5 and 3.5 weeks of age and analyzed at 5.5 weeks of age. The *dy*^*2J*^*/dy*^*2J*^ quadriceps muscle displayed an increased number of small muscle fibers compared with wild-type muscle (S1 Fig). Bortezomib administration in wild-type mice significantly increased the proportion of small muscle fibers (cross-sectional area 1–1000 μm^2^) and reduced the proportion of large fibers (cross-sectional area 1500–2500 μm^2^) (S1 Fig). However, bortezomib administration did not alter fiber size distribution in *dy*^*2J*^*/dy*^*2J*^ quadriceps muscle (S1 Fig). A significantly increased number of muscle fibers with centrally located nuclei was noticed in 5.5-week-old *dy*^*2J*^*/dy*^*2J*^ quadriceps muscle compared to wild-type muscle, but central nucleation was not affected by bortezomib treatment (regardless of genotype) (S1 Fig). Laminin α2 chain-deficiency is also characterized by pathological fibrosis and *dy*^*2J*^*/dy*^*2J*^ quadriceps muscle at 5.5 weeks of age displayed increased fibrosis compared with wild-type (roughly 2.5-fold) as shown by increased tenascin-C deposition and Masson’s trichrome staining. Yet, two injections of bortezomib administration did not reduce fibrosis in *dy*^*2J*^*/dy*^*2J*^ quadriceps muscle (S1 Fig). Finally, we analyzed the body weight. It was only mildly reduced in 5.5-week-old *dy*^*2J*^*/dy*^*2J*^ mice compared to wild-type animals (reduction was not statistically significant) and bortezomib administration had no significant impact on body weight, regardless of genotype (S1 Fig). In summary, two bortezomib injections in *dy*^*2J*^*/dy*^*2J*^ mice at 2.5 and 3.5 weeks of age did not have any beneficial effects on muscle morphology.

### Six bortezomib injections do not reduce muscular dystrophy in *dy*^*2J*^*/dy*^*2J*^ mice

In order to analyze whether additional injections could be advantageous, we tested the following bortezomib injection regimen: three i.v. injections at 2.5, 3.5 and 4.5 weeks of age and three s.c. injections at 5.5, 6.5 and 7.5 weeks of age and final analysis at 8.5 weeks of age. We started with 0.4 mg/kg and then gradually decreased the dose to 0.16 mg/kg in order to avoid serious adverse effects of bortezomib. Still, it has been demonstrated that bortezomib significantly attenuates the severity of collagen-induced arthritis (another musculoskeletal disorder) in mice within this dose range [15]. Also, in several previous studies it has been demonstrated that bortezomib is taken up in skeletal muscle after intravenous injections in mice and rats [16–18].

**Fig 1:**
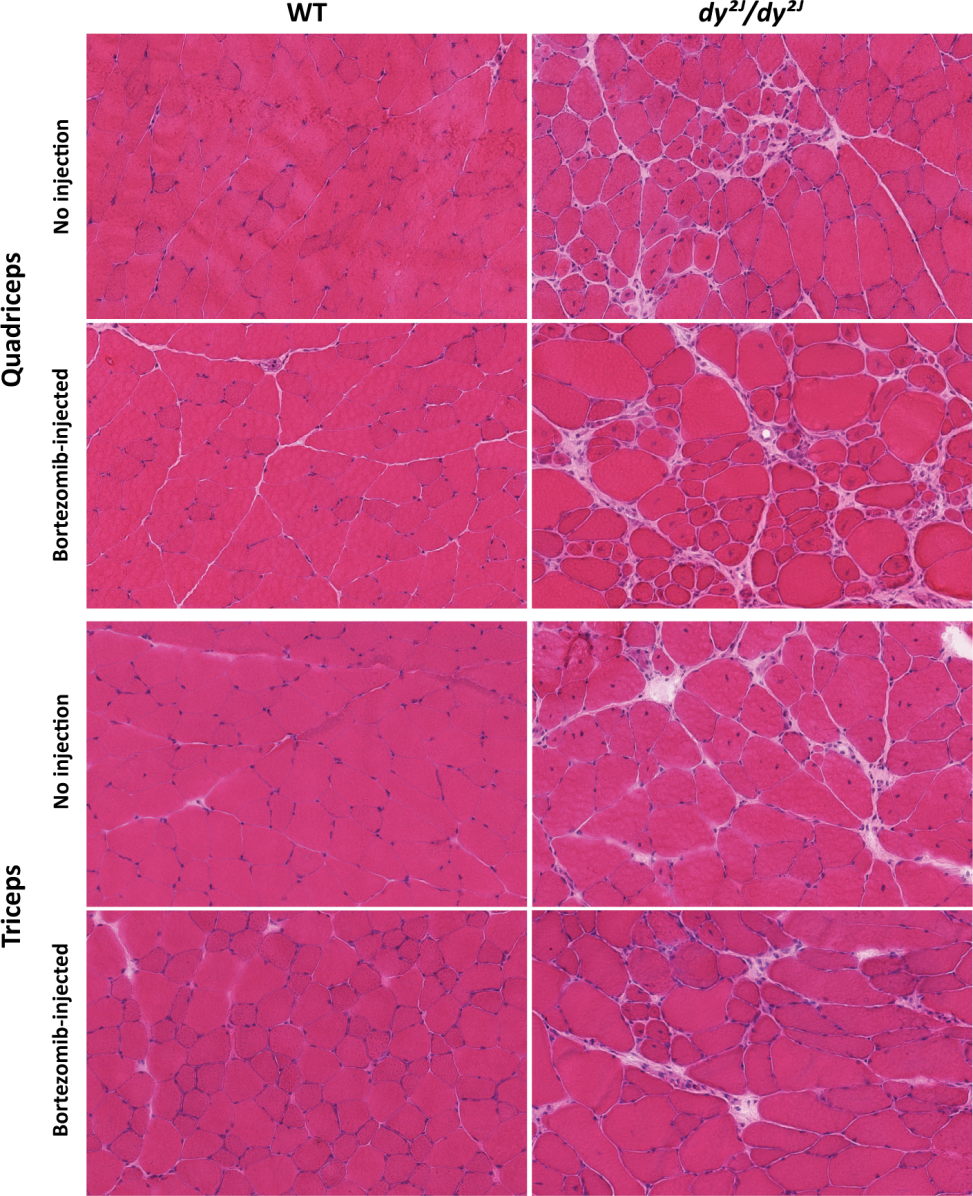
Bortezomib treatment does not improve the muscle morphology of *dy*^*2J*^*/dy*^*2J*^ quadriceps and triceps muscle. Hematoxylin and eosin staining of cross-sections of quadriceps and triceps muscle from wild-type (WT), bortezomib-treated WT, *dy*^*2J*^*/dy*^*2J*^ and bortezomib-treated *dy*^*2J*^*/dy*^*2J*^ mice (8.5-week-old) revealed myopathic changes (muscle degeneration/regeneration, fiber size variability and connective tissue infiltration) in both *dy*^*2J*^*/dy*^*2J*^ and bortezomib-treated *dy*^*2J*^*/dy*^*2J*^ mice. Magnification x 8.6.

**Fig 2:**
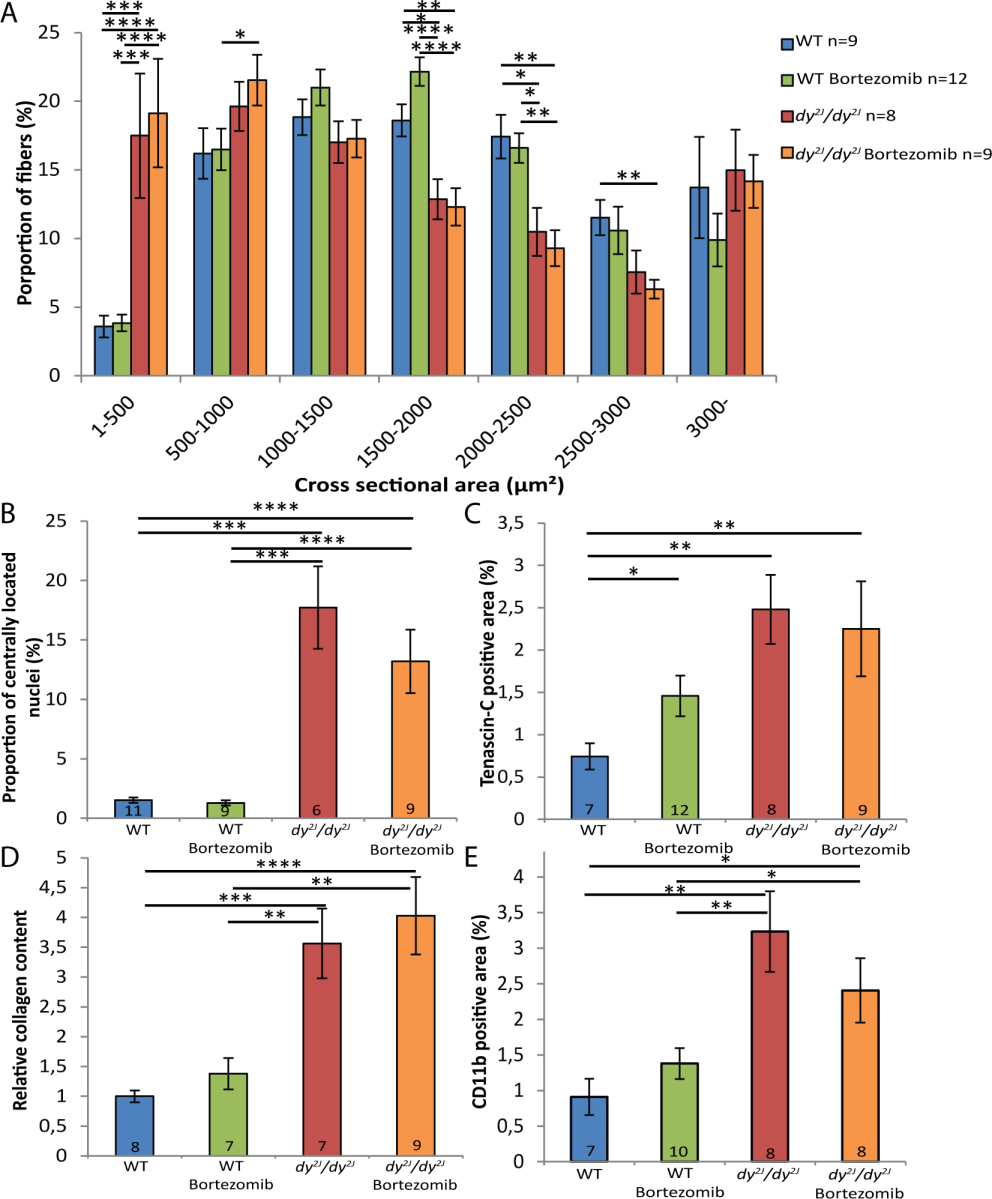
Bortezomib treatment does not normalize fiber size distribution or reduce fibrosis and inflammation in *dy*^*2J*^*/dy*^*2J*^ quadriceps muscle. (A) In *dy*^*2J*^*/dy*^*2J*^ mice, a higher proportion of muscle fibers had small diameters. The fiber-size distribution was not changed toward the values of wild-type quadriceps muscle in the bortezomib-injected *dy*^*2J*^*/dy*^*2J*^ mice. (B) Bortezomib did not affect the number of centrally nucleated fibers in quadriceps muscle of *dy*^*2J*^*/dy*^*2J*^ mice. (C) Bortezomib did not decrease the tenascin-C positive area in quadriceps muscle of *dy*^*2J*^*/dy*^*2J*^ animals. (D) The relative collagen content in quadriceps muscle was not changed in bortezomib-treated *dy*^*2J*^*/dy*^*2J*^ mice. (E) Bortezomib did not significantly reduce the amount of CD11b-positive immune cells in *dy*^*2J*^*/dy*^*2J*^ quadriceps muscle. Data are expressed as means ± SEM. Number of mice analyzed is indicated in data bars. * *P* < 0.05; ** *P* < 0.01; *** *P* < 0.001; **** *P* < 0.0001.

**Fig 3:**
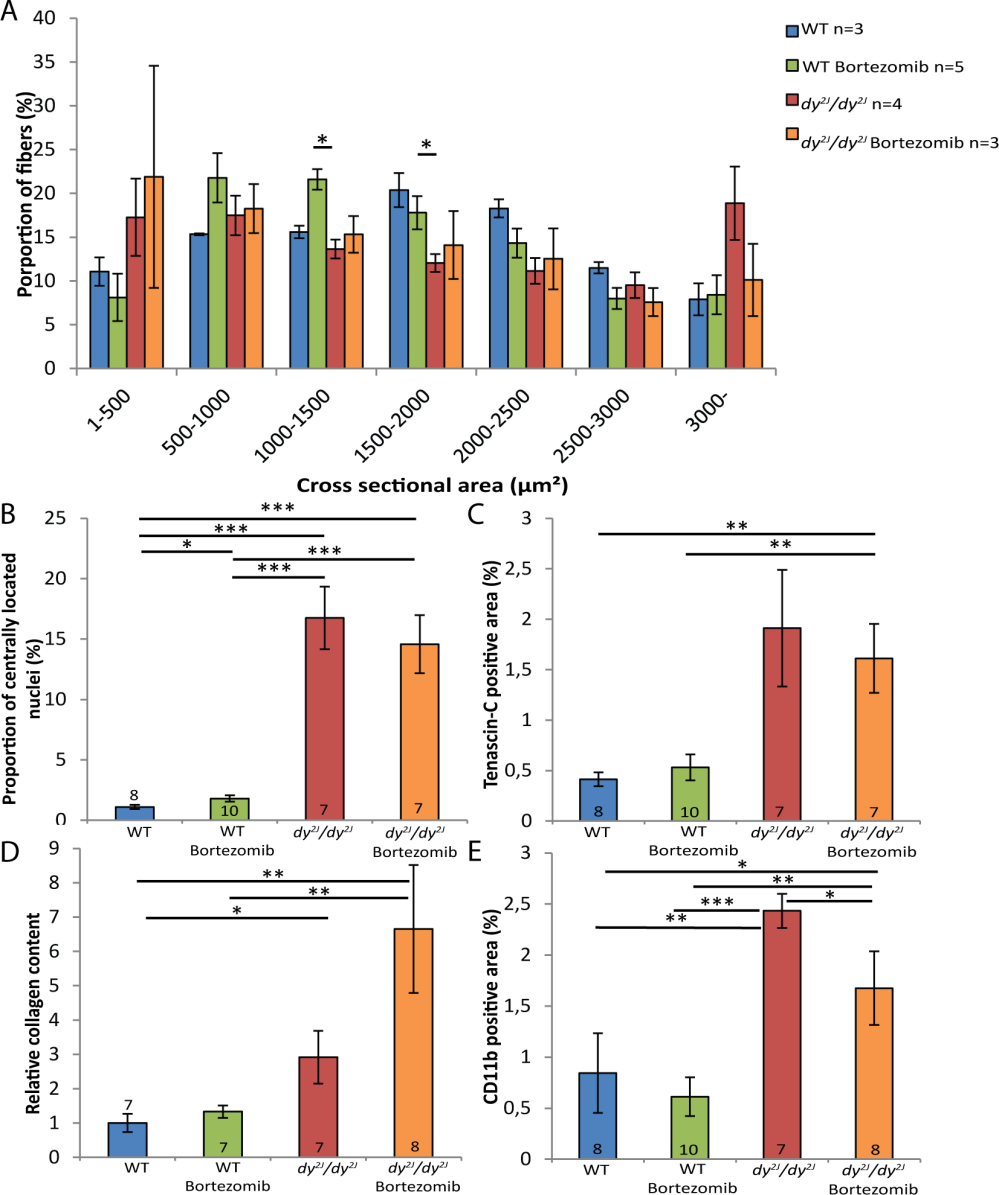
Bortezomib treatment does not normalize fiber size distribution or reduce fibrosis in *dy*^*2J*^*/dy*^*2J*^ triceps muscle. (A) In *dy*^*2J*^*/dy*^*2J*^ mice, a higher proportion of muscle fibers had small diameters. The fiber-size distribution was not changed toward the values of wild-type triceps muscle in the bortezomib-injected *dy*^*2J*^*/dy*^*2J*^ mice. (B) Bortezomib did not affect the number of centrally nucleated fibers in triceps muscle of *dy*^*2J*^*/dy*^*2J*^ mice. (C) Bortezomib did not decrease the tenascin-C positive area in triceps muscle of *dy*^*2J*^*/dy*^*2J*^ animals. (D) The relative collagen content in triceps muscle was not significantly altered in bortezomib-treated *dy*^*2J*^*/dy*^*2J*^ mice. (E) Bortezomib slightly reduced the amount of CD11b-positive immune cells in *dy*^*2J*^*/dy*^*2J*^ triceps muscle. Data are expressed as means ± SEM. Number of mice analyzed is indicated in data bars. * *P* < 0.05; ** *P* < 0.01; *** *P* < 0.001; **** *P* < 0.0001.

One hindlimb (quadriceps femoris) and one forelimb (triceps brachii) muscle were chosen for histological analyses. We found that the muscle morphology of *dy*^*2J*^*/dy*^*2J*^ muscle was not further improved by additional injections of bortezomib. Dystrophic changes such as fiber size variability, central nucleation and connective tissue infiltration were evident to a similar degree in quadriceps and triceps muscle of untreated and bortezomib-treated *dy*^*2J*^*/dy*^*2J*^ mice (Fig 1). In 8.5-week-old *dy*^*2J*^*/dy*^*2J*^ animals, the quadriceps and triceps muscle fiber-size distribution was shifted to smaller diameters compared to wild-type muscles. However, bortezomib administration did not affect fiber size distribution in either *dy*^*2J*^*/dy*^*2J*^ quadriceps or triceps muscle (Figs 2A and 3A). An increased number of muscle fibers with centrally located nuclei was also noticed in 8.5-week-old *dy*^*2J*^*/dy*^*2J*^ quadriceps and triceps muscle compared with wild-type muscles but again, central nucleation was not affected by bortezomib treatment (Figs 2B and 3B). We evaluated fibrosis with two independent methods. We found significantly increased tenascin-C expression in *dy*^*2J*^*/dy*^*2J*^ quadriceps muscle compared with wild-type muscle and a trend for enhanced tenascin-C expression in *dy*^*2J*^*/dy*^*2J*^ triceps muscle. Nonetheless, there was no reduction of tenascin-C expression in quadriceps and triceps muscle of bortezomib-treated *dy*^*2J*^*/dy*^*2J*^ mice (Figs 2C and 3C). We also used a biochemical collagen quantification assay, which revealed significantly increased collagen content (approximately 2–3-fold) in *dy*^*2J*^*/dy*^*2J*^ in quadriceps and triceps muscle. This increase did not change upon administration of bortezomib to *dy*^*2J*^*/dy*^*2J*^ animals (Figs 2D and 3D). Since inflammation is a feature of MDC1A, we assessed the inflammatory response in treated and non-treated muscle. We observed a significant upregulation of CD11b-positive cells (monocytes/macrophages) in *dy*^*2J*^*/dy*^*2J*^quadriceps and triceps muscle. Bortezomib did not reduce the number of CD11b-positive immune cells in quadriceps muscle, but there was a slight but significant reduction in triceps muscle (Figs 2E and 3E).

**Fig 4:**
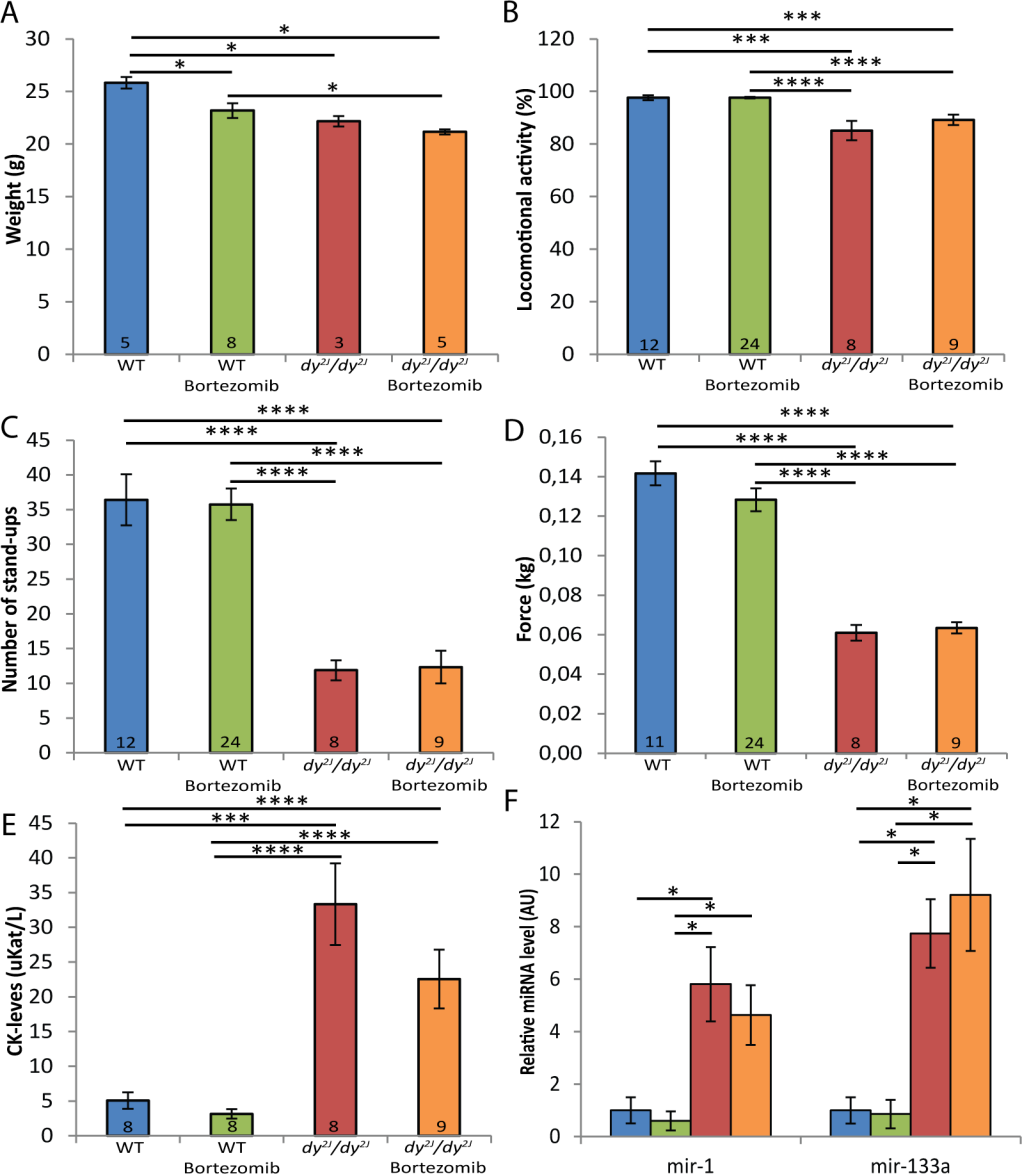
Bortezomib treatment does not increase weight, locomotion, muscle strength or reduce plasma levels of CK and miRNA. (A) The body weight was significantly reduced in *dy*^*2J*^*/dy*^*2J*^ male mice and bortezomib-treated wild-type and *dy*^*2J*^*/dy*^*2J*^ male mice compared to wild-type mice. (B) Administration of bortezomib did not improve the exploratory locomotion of *dy*^*2J*^*/dy*^*2J*^ animals in an open-field test. (C) Bortezomib did not increase the number of stand-ups in *dy*^*2J*^*/dy*^*2J*^ mice. (D) Grips strength testing revealed no increase in fore-limb muscle strength in bortezomib-treated *dy*^*2J*^*/dy*^*2J*^ mice. (E) Analysis of CK levels in plasma revealed a significant increase in CK levels in *dy*^*2J*^*/dy*^*2J*^mice, which was not significantly reduced by bortezomib. (F) RT-qPCR analysis showed enriched plasma levels of miR-1 and miR-133a in *dy*^*2J*^*/dy*^*2J*^ animals, which were not decreased by bortezomib. Blue bar represents wild-type mice; green bortezomib-treated wild-type; red *dy*^*2J*^*/dy*^*2J*^; orange bortezomib-treated *dy*^*2J*^*/dy*^*2J*^. Data are expressed as means ± SEM. Number of mice analyzed is indicated in data bars, except in panel F, in which n = 4. * *P* < 0.05; ** *P* < 0.01; *** *P* < 0.001; **** *P* < 0.0001.

Next, we assessed the overall health status of bortezomib-treated *dy*^*2J*^*/dy*^*2J*^ animals by investigating if bortezomib injections contributed to increased body weight, improved locomotive behavior and enhanced muscle strength. The body weight was significantly reduced in 8.5-week-old *dy*^*2J*^*/dy*^*2J*^ male mice compared to wild-type male mice and bortezomib did not enhance the body weight of male *dy*^*2J*^*/dy*^*2J*^ mice. Instead, multiple injections of bortezomib decreased the body weight (approximately 10%) of male wild-type mice compared with untreated wild-type male mice (Fig 4A). In female wild-type mice, on the other hand, multiple injections of bortezomib did not cause any weight loss (data not shown). We have previously demonstrated that exploratory locomotion of *dy*^*3K*^*/dy*^*3K*^ mice in an open field test is significantly reduced compared to wild-type animals [9, 10]. Similarly, *dy*^*2J*^*/dy*^*2J*^ mice at the age of 8.5 weeks were also significantly less active (roughly 1.1-fold) compared to wild-type mice. Bortezomib did, however, not enhance the exploratory locomotion of *dy*^*2J*^*/dy*^*2J*^ mice (Fig 4B). We also analyzed the average number of stand-ups over a 5-minute period and observed that 8.5-week-old *dy*^*2J*^*/dy*^*2J*^ mice displayed a significant reduction (around 3-fold) in stand-up activity compared to wild-type mice. No increase in stand-up activity was noted upon bortezomib treatment (Fig 4C). Subsequently, we measured the muscle strength of *dy*^*2J*^*/dy*^*2J*^ and wild-type forelimbs using a grip-strength meter. *Dy*^*2J*^*/dy*^*2J*^ mice were significantly weaker (approximately 2.3-fold) compared to control mice and bortezomib did not increase the grip strength (Fig 4D).

Lastly, we measured plasma creatine kinase (CK) levels and the expression of two muscle-specific miRNAs in plasma from 8.5-week-old animals in order to examine the sarcolemmal integrity of skeletal muscle fibers. A significant increase in CK levels was observed in *dy*^*2J*^*/dy*^*2J*^ mice but bortezomib did not significantly lower the CK levels in the blood (Fig 4E). We have recently demonstrated that the expression of miR-1 and miR-133a is significantly increased in plasma from both *dy*^*3K*^*/dy*^*3K*^ and *dy*^*2J*^*/dy*^*2J*^ mice. More importantly, administration of bortezomib resulted in a partial normalization of plasma levels of miR-1 and miR-133a in *dy*^*3K*^*/dy*^*3K*^ mice [12]. However, bortezomib failed to reduce miR-1 and miR-133a plasma levels in *dy*^*2J*^*/dy*^*2J*^ mice (Fig 4F).

In this study we demonstrated that bortezomib treatment has no significant beneficial effects in *dy*^*2J*^*/dy*^*2J*^ mice. Bortezomib failed to improve the muscle phenotype as measured by histological and functional assays. These data are in contrast to our previous reports in which we found that proteasome inhibitors MG-132 and bortezomib partially improved the phenotype of *dy*^*3K*^*/dy*^*3K*^mice, which display a more severe phenotype compared to *dy*^*2J*^*/dy*^*2J*^ animals [8–10]. The lack of beneficial effects was somewhat surprising considering that proteasome activity appears augmented in *dy*^*2J*^*/dy*^*2J*^ muscle, but it should be noted that while the expression of proteasome-related genes (e.g. MuRF-1, atrogin-1 and ubiquitin) is increased more than 10-fold in *dy*^*3K*^*/dy*^*3K*^ muscle it is only increased 1.5–2-fold in *dy*^*2J*^*/dy*^*2J*^ muscle. Also, the general muscle wasting is not extensive in *dy*^*2J*^*/dy*^*2J*^ mice as the body weight is only slightly decreased at 5.5 and 8.5 weeks of age. This is in sharp contrast to *dy*^*3K*^*/dy*^*3K*^ mice that are severely emaciated at 3–4 weeks of age [8–10, 19].

Another possibility for the absence of beneficial effects in *dy*^*2J*^*/dy*^*2J*^ animals is that *dy*^*2J*^*/dy*^*2J*^ and *dy*^*3K*^*/dy*^*3K*^ mice may require different dosing of bortezomib. We previously treated *dy*^*3K*^*/dy*^*3K*^ mice with 0.4 mg/kg bortezomib as 0.8 mg/kg (previously administered to *mdx* mice [18]) did not improve lifespan of *dy*^*3K*^*/dy*^*3K*^ mice [10]. Similarly, we first administrated 0.8 mg/kg in two *dy*^*2J*^*/dy*^*2J*^ mice but these mice died shortly after injection (note that *dy*^*2J*^*/dy*^*2J*^ mice have a life span of several months) (data not shown) and therefore we subsequently injected 0.4 mg/kg, followed by lower doses. Still it is possible, but not very likely, that 0.5–0.7 mg/kg could have been advantageous in *dy*^*2J*^*/dy*^*2J*^ mice.

In conclusion, these findings do not support a putative role for bortezomib in the treatment of MDC1A with partial laminin α2 chain-deficiency. However, we would like to stress that proteasome inhibition (two injections of bortezomib) diminished the severity of muscle dysfunction in *dy*^*3K*^*/dy*^*3K*^ mice [9, 10] but we have not yet evaluated the effects of multiple injections in *dy*^*3K*^*/dy*^*3K*^ mice. Nevertheless, it is plausible that proteasome inhibition could be a useful supportive therapy in patients with complete deficiency of laminin α2 chain.

## Supporting Information

S1 Fig Two bortezomib injections do not improve muscle morphology, reduce fibrosis or increase body weight in *dy*^*2J*^*/dy*^*2J*^ mice. (A) The shift in fiber-size distribution was not affected by administration of bortezomib to *dy*^*2J*^*/dy*^*2J*^ mice. (B) Bortezomib did not affect the number of centrally nucleated fibers in quadriceps muscle of *dy*^*2J*^*/dy*^*2J*^ mice. (C) Bortezomib did not decrease the tenascin-C positive area in quadriceps muscle *dy*^*2J*^*/dy*^*2J*^ animals. (D) Masson’s trichrome staining of transverse cryosections of *dy*^*2J*^*/dy*^*2J*^ quadriceps muscle did not reveal reduced collagen content upon bortezomib treatment. (E) The body weight did not change between genotypes. Data are expressed as means ± SEM. Number of mice analyzed is indicated in data bars. * *P* < 0.05; ** *P* < 0.01; *** *P* < 0.001.
(TIF)
