# Supplementary figures and images for: Bortezomib Does Not Reduce Muscular Dystrophy in the dy2J/dy2J Mouse Model of Laminin α2 Chain-Deficient Muscular Dystrophy
Source: PLoS One. 2016 Jan 5;11(1):e0146471. doi: 10.1371/journal.pone.0146471 (PMC4701230; doi:10.1371/journal.pone.0146471)

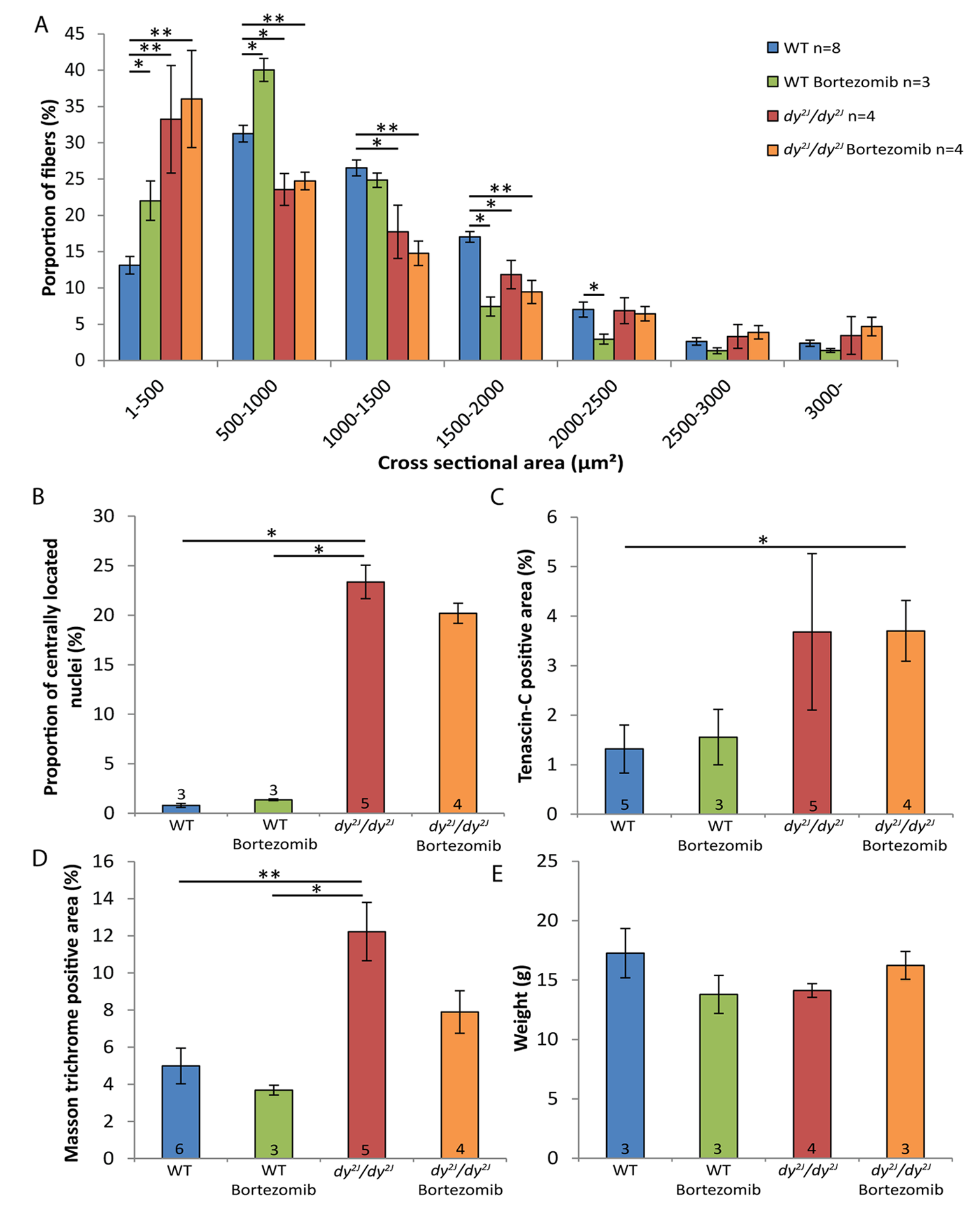

Supplement: S1 Fig — (A) The shift in fiber-size distribution was not affected by administration of bortezomib to dy2J/dy2J mice. (B) Bortezomib did not affect the number of centrally nucleated fibers in quadriceps muscle of dy2J/dy2J mice. (C) Bortezomib did not decrease the tenascin-C positive area in quadriceps muscle dy2J/dy2J animals. (D) Masson’s trichrome staining of transverse cryosections of dy2J/dy2J quadriceps muscle did not reveal reduced collagen content upon bortezomib treatment. (E) The body weight did not change between genotypes. Data are expressed as means ± SEM. Number of mice analyzed is indicated in data bars. * P < 0.05; ** P < 0.01; *** P < 0.001. (TIF) [file pone.0146471.s001.tif]
